# Analysis of insecticides in long-lasting insecticidal nets using X-ray fluorescence spectroscopy and correlation with bioefficacy

**DOI:** 10.3389/fpara.2023.1258429

**Published:** 2023-10-11

**Authors:** Melanie Koinari, Nakei Bubun, David Wilson, Evodia Anetul, Lincoln Timinao, Petrina Johnson, Norelle L. Daly, Moses Laman, Tim Freeman, Stephan Karl

**Affiliations:** ^1^ Australian Institute of Tropical Health and Medicine, James Cook University, Smithfield, QLD, Australia; ^2^ Vector-borne Disease Unit, Papua New Guinea Institute of Medical Research, Madang, Madang, Papua New Guinea; ^3^ Malaria Control Program, Rotarians Against Malaria Papua New Guinea, Port Moresby, Papua New Guinea

**Keywords:** XRF, liquid chromatography-mass spectroscopy (LC-MS), bednets, insecticide content, LLIN, ITNs, deltamethrin, alpha cypermethrin

## Abstract

**Background:**

Long-lasting insecticidal nets (LLINs) are a key vector control tool used for the prevention of malaria. Active ingredient (AI) measurements in LLINs are essential for evaluating their quality and efficacy. The main aim of the present study was to determine the utility of X-ray fluorescence (XRF) spectroscopy as a suitable field-deployable tool for total AI quantification in LLINs.

**Methods:**

New and unused LLIN samples containing deltamethrin (PermaNet® 2.0, *n* = 35) and alpha-cypermethrin (SafeNet®, *n* = 43) were obtained from batches delivered to Papua New Guinea (PNG) for mass distribution. Insecticides were extracted from the LLINs using a simple extraction technique and quantified using liquid chromatography mass spectrometry (LC-MS). The LC-MS results were correlated with XRF spectroscopy measurements on the same nets. Operators were blinded regarding the type of net. Bioefficacy of the LLIN samples was tested using WHO cone bioassays and test results were correlated with total AI content.

**Results:**

The results indicate correlation between quantitative XRF and LC-MS. Interestingly, the total AI content was negatively correlated with bioefficacy in PermaNet® 2.0 (especially in recently manufactured nets). In contrast, AI content was positively correlated with bioefficacy in SafeNet®. These results indicate that the chemical content analysis in predelivery inspections does not always predict bioefficacy.

**Conclusion:**

XRF is a promising field-deployable tool for quantification of both deltamethrin- and alpha-cypermethrin-coated LLINs. Because total AI content is not always a predictor of the efficacy of LLINs to kill mosquitoes, bioefficacy measurements should be included in predelivery inspections.

## Background

Hundreds of millions of long-lasting insecticidal nets (LLINs) for vector control are procured annually by international donors and distributed (Bhatt et al., 2015). LLIN distributions have helped to reduce the malaria burden in many endemic countries (WHO, 2017). Despite this, and the target to eliminate malaria globally, malaria case numbers have stagnated in the last decade (WHO, 2021). Mass distribution of LLINs is the backbone of malaria vector control in Papua New Guinea (PNG). About 14 million bed nets have been distributed in PNG since 2009 (Alliance for Malaria Prevention, 2022). Bed nets are distributed every 3 years to all villages in PNG below 1,600 meters, following a “rolling” distribution schedule. Previous studies in PNG found that bed net performance significantly decreased in 2013 following a manufacturing change involving the chemical coating of the nets, leading to reduced efficacy of the nets to kill mosquitoes and thus reduced community protection (Vinit et al., 2020; Bubun et al., 2021; Bubun et al., 2022).

Many previous studies have shown that bioefficacy in used LLINs is strongly correlated with total insecticide content (Kweka et al., 2011; Anshebo et al., 2014; Katusele et al., 2014). This is expected because when nets are washed or exposed to UV radiation (sun exposure), total active ingredient (AI) content decreases and once a threshold AI concentration is reached, the 100% 24-h mortality normally observed with fully susceptible strains in new and unwashed nets is not maintained. However, other studies have demonstrated that recently manufactured new and unused LLIN products may not be able to fulfil WHO cone bioassay performance criteria within susceptible mosquito colonies, even though their total AI content was determined to be adequate in predelivery inspections (Vinit et al., 2020; MBwambo et al., 2022). This may be for different reasons, including the restricted bioavailability of AIs on the net surface due to the nature of the LLIN coating or the presence of AIs in chemical or physical states that may be detrimental to their effectiveness (such as isomers with reduced potency or crystalline states that may reduce the uptake of AI by mosquitoes) (Maguire, 1990; Yang et al., 2020; Bubun et al., 2021; Bubun et al., 2022). Thus, it is important to fully understand the relationship between LLIN bioefficacy, as determined in simple and standardized evaluations, e.g., using WHO cone bioassays, and total AI concentrations and presentations on the surface of LLINs.

Chemical analyses of the LLINs form an integral part of the WHO guidelines for testing new nets and also in monitoring the durability of LLINs under operational conditions (WHO, 2013). As stated in the guidelines, the insecticide content of a net sample should be analyzed in accordance with the methods published by the Collaborative International Pesticides Analytical Council (CIPAC), specifically high-pressure liquid chromatography (HPLC) (CIPAC, 2021a; CIPAC, 2021b; CIPAC, 2021c). However, laboratories in malaria-endemic developing countries that evaluate LLINs for malaria control and prevention or conduct quality assurance tests for national programs may not have easy access to the equipment needed or the expertise required. Therefore, simple, in-field approaches to quantify insecticides in LLIN products would be useful. Several alternative methods have been developed to quantify pyrethroids in LLINs, including rapid colorimetric field tests and various quantitative and imaging techniques (Green et al., 2009; Jenkins et al., 2013; Smith et al., 2018).

A promising new alternative method for AI quantification is X-ray fluorescence (XRF) spectroscopy. Researchers in Ghana, Ethiopia, and Guatemala have demonstrated the utility of XRF for fast quantification of deltamethrin in the field, specifically for durability assessments of PermaNet® 2.0 LLINs after 3–38 months of use (Smith et al., 2007; Anshebo et al., 2014; Castellanos et al., 2021). While XRF has been shown to be able to quantify deltamethrin in LLINs, there are limited reports on its application for other insecticides, for example in alpha cypermethrin-coated LLINs, where theoretically this should also be possible.

The present study had two main aims. Firstly, to validate a field-based method (XRF spectroscopy) for both deltamethrin- and alpha-cypermethrin-coated LLINs and to correlate total AI content measured with XRF with a laboratory-based method [liquid chromatography mass spectrometry (LC-MS)]. The correlation between these techniques will help to determine the utility of XRF as a suitable field-deployable tool for total AI quantification for both insecticides, which are found in the vast majority of prequalified LLIN products. Secondly, to determine the correlation between total AI content and bioefficacy in two LLIN products (one deltamethrin product and one alpha-cypermethrin product) delivered to PNG for mass distribution in the new and unused state.

## Methods

WHO cone bioassays and XRF spectroscopy were conducted at the Vector-borne Diseases Unit of the Papua New Guinea Institute of Medical Research (PNG IMR), while the LC-MS was conducted at the Australian Institute of Tropical Health and Medicine (AITHM), James Cook University (JCU).

### LLIN sampling

PermaNet® 2.0 (*n* = 35) and SafeNet® (*n* = 43) LLINs, all unused and in their original packaging, were included in the present study. As described by Vinit et al., 2020 (Vinit et al., 2020), unused PermaNet® 2.0 LLINs manufactured between 2007 and 2017 were obtained from villages or provincial health authorities in various provinces in PNG. Rotarians Against Malaria (RAM) PNG provided PermaNet® 2.0 LLINs manufactured in 2018 and 2019 from consignments dedicated to different provinces (Vinit et al., 2020). SafeNet® LLINs manufactured in 2019 and 2020 were provided by RAM PNG. Details of the selected nets can be found in **Supplementary Table S1**.

Pairs of samples were cut from adjacent positions. One net piece per position was sent to JCU and the second, adjacent piece was retained in PNG for testing. The project investigators and facility technicians were blinded to the identity of the products until the end of the study. After all experiments were completed, data from the PNG IMR cone bioassays and XRF were sent to JCU, and the blinding was disclosed to the JCU investigators to match the results with the type of study net to enable analysis.

### X-ray fluorescence analysis on single layers of netting

Proxy element (bromine and chlorine) content was measured in a monolayer of netting, using a Vanta® Handheld XRF Analyser (Olympus, Australia). To increase the limit of detection, the instrument was used with a shielded measurement chamber and a silica standard background, also sourced from Olympus. Bromine and chlorine were quantified by the built-in software and expressed in parts-per-million (ppm). A measurement took about 45 s.

The stand and the shielded chamber, together with the silica background used in this study, are very small and portable and could easily be moved to any “true” field location (**Supplementary Figure S1**). The measurements could have been taken without the chamber; however, using the chamber and the silica background potentially provided better sensitivity and less interference.

### Liquid chromatography mass spectrometry

#### Insecticide extraction

LC-MS analysis was performed on methanol extracts of two samples of each net (each piece was 2 cm × 2 cm), which were extracted separately in duplicate to generate technical and biological replicates. Net samples were weighed and submerged in 1 mL of LC-MS-grade methanol (Merck, Australia) in individual 2.0 mL Eppendorf Safelock tubes (Eppendorf, Australia) that had previously been shown as being free from any contaminants (e.g., plasticizer from manufacture). The tube was placed on a vortex for 60 s then briefly centrifuged.

#### LC-MS measurement

LC-MS was performed using a Shimadzu LC-MS2020 (Shimadzu, Japan) and a Phenomenex Lux cellulose-2 chiral column (150 mm × 2.0 mm; Phenomenex, Australia) at 40°C, and a 5 mM ammonium acetate/water (Solvent A) and 100% methanol (Solvent B; LiChrosolv LC-MS-grade, Merck) mobile phase at 0.25 mL/min flow rate. Samples (20 µL) were eluted isocratically in 82% Solvent B over 12 min and UV absorbance monitored at 246 nm and 280 nm. Mass spectra were collected in positive ion modes over a scan range of *m/z* 200–800 with a detector voltage of 1.15 kV, a nebulizing gas flow of 1.5 L/min, and a drying gas flow of 3.0 L/min.

#### Standards

Analytical grade samples of deltamethrin and alpha-cypermethrin, to be used as standards, were purchased from Merck (Australia). Standard curves that spanned the anticipated insecticide concentration range for each LLIN product (for PermaNet® 2.0 LLINs the target deltamethrin concentration is 1.8 g/kg and for SafeNet® LLINs the target concentration is 5g/kg alpha-cypermethrin) were created by preparing dilution series of known insecticide concentrations in methanol. A primary stock solution was prepared by dissolving an accurately weighed amount of insecticide on an analytical balance (Mettler Toledo, Australia) in 1 mL of methanol. The stock was then diluted to five times the expected target concentration, followed by a 1.5-fold dilution series for a total of 10 dilution samples of the standard insecticide. These known concentrations of standard insecticides were included in every LC-MS analysis of net samples (with unknown insecticide content).

#### Extraction protocol validation

To determine the extraction efficiency of the insecticides from the nets, and the potential impact of solvent volume and extraction time changes, a subset of net samples was subjected to the following validation experiments. Firstly, for solvent volume experiments, *n* = 7 samples cut from each LLIN (2012-PermaNet® 2.0, *n* = 2) were submerged in 500 µL, 750 µL, 1,000 µL, 1,200 µL, 1,500 µL, 1,750 µL, and 2,000 µL of methanol. Extracts from each volume were analyzed by LC-MS in triplicate. Secondly, for extraction time, *n* = 5 samples cut from each LLIN (2012-PermaNet® 2.0, *n* = 2) were each extracted in 1,000 µL of methanol. The five samples were placed in the solvent at the same time but were removed after 10 min (0 h), 1 h, 3 h, 5 h, and 24 h. Extracts from each extraction time point were analyzed by LC-MS in triplicate. Thirdly, to validate complete extraction from the net samples, *n* = 2 samples were cut from each LLIN (SafeNet® 2019, *n* = 1 and SafeNet® 2020, *n* = 1) and each piece of net was subjected to two consecutive rounds of extraction under the following conditions: solvent volume = 1,000 µL, extraction time = 10 min, vortex = 1 min, and a brief centrifuge.

### WHO cone bioassays

Cone bioassays were performed following the WHO guidelines and under laboratory conditions with 28.5°C ± 2.7°C and 65% ± 9% relative humidity (WHO, 2013). Using an aspirator, five insecticide susceptible, non-blood-fed, 2- to 5-day-old female *Anopheles farauti* mosquitoes were introduced into a cone and a cotton ball was used to plug the hole. Mosquitoes were exposed to the net pieces for 3 min (timed individually for each cone), after which they were gently transferred from the cones to a holding cup screened with untreated netting and provided access to 10% sugar solution via a soaked piece of cotton wool placed on top of the netting. The number of mosquitoes knocked down after 60 min and dead at 24 h after exposure were recorded. LLINs that caused ≥ 95% 60-min knockdown or ≥ 80% 24-h mortality were regarded as meeting the WHO efficacy criteria.

### Data analysis

**Figure 1:**
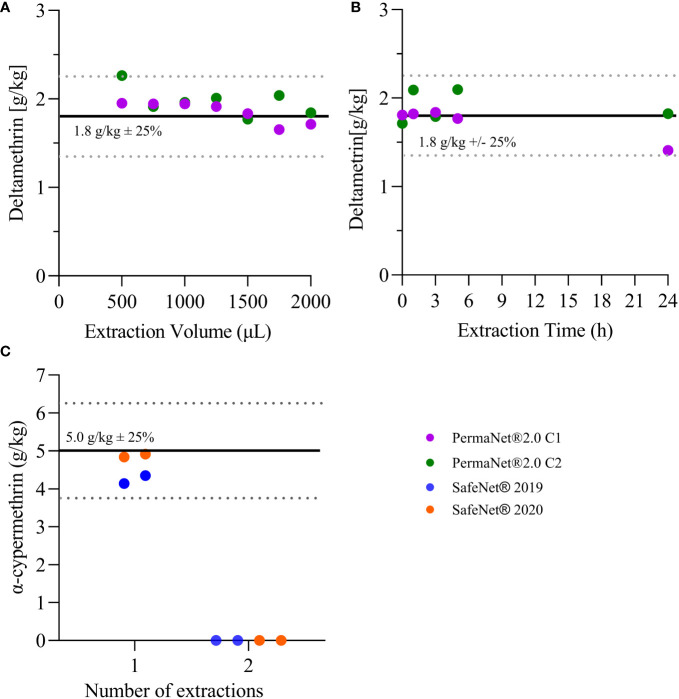
Extraction efficiency. **(A)** Shows the concentration of deltamethrin for *n* = 7 samples (from each 2012-PermaNet® 2.0 LLIN) extracted at various volumes of methanol. **(B)** Shows the concentration of deltamentrin for the *n* = 5 samples (from each 2012-PermaNet® 2.0 LLIN) extracted at different time points. **(C)** Shows the concentration of alpha-cypermethrin for the *n* = 4 samples (from 2019- and 2020-SafeNet® LLINs), which were extracted at 10 min in 1 mL of methanol. Horizontal lines indicate the target AI concentrations as per product label (average ± 25%).

**Figure 2:**
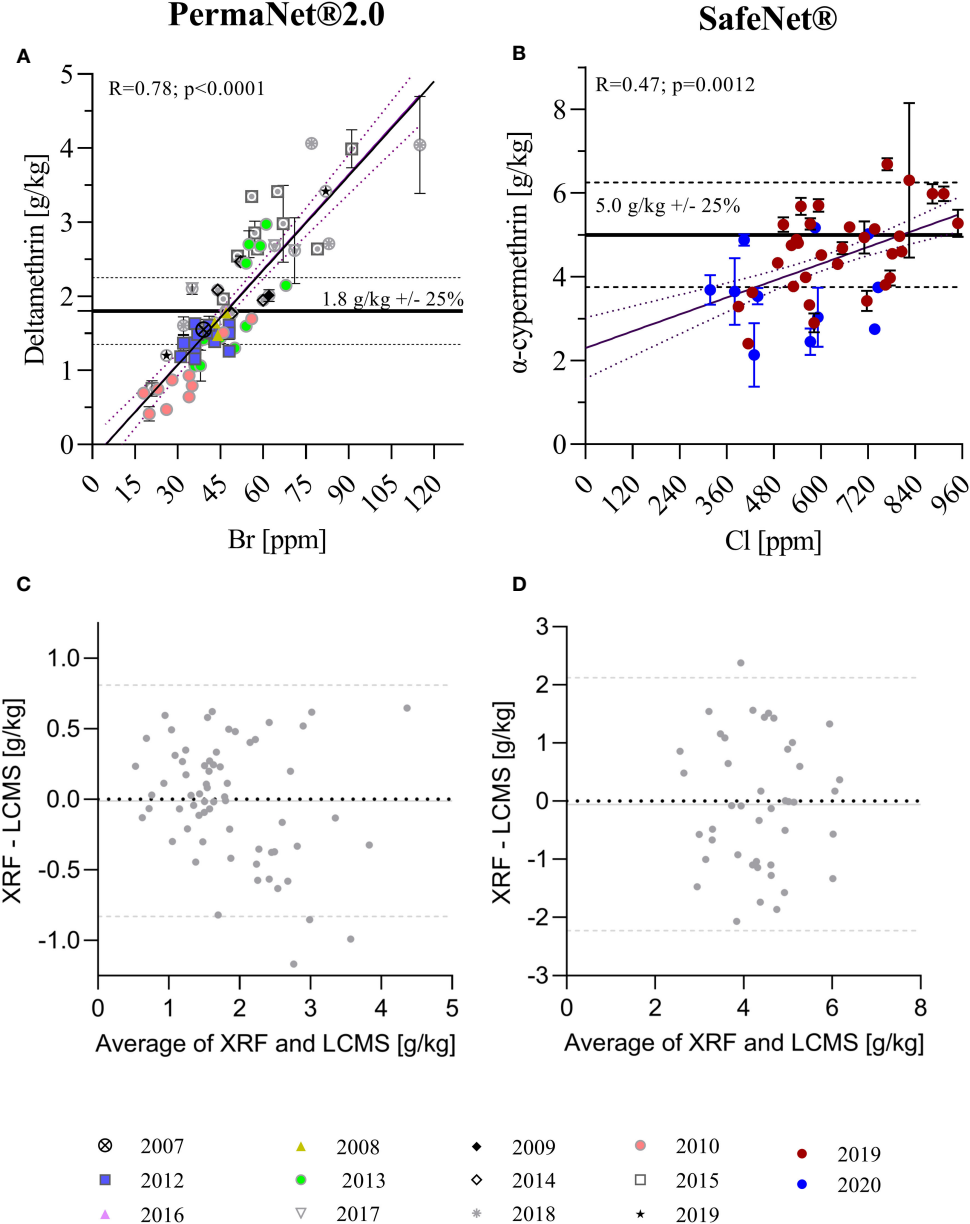
Correlation of total AI quantification using XRF and LC-MS techniques. **(A)** Correlation of XRF (in ppm) and LC-MS (in g/kg) data for PermaNet® 2.0 samples with manufacturing years between 2007 and 2019. **(B)** Correlation of XRF and LC-MS data for SafeNet® samples with manufacturing years 2019 and 2020. Horizontal lines in **(A, B)** indicate the target concentrations for the products as per product label. Simple linear regression curves and 95% confidence bands are also shown. The regression was used to convert the XRF data in ppm to the corresponding g/kg. **(C, D)** Bland–Altman analyses for XRF versus LC-MS quantification of total AI content [**(C)** – PermaNet® 2.0 LLIN, **(D)** – SafeNet® LLIN]. The plots show the differences XRF/LC-MS data pairs over their averages. Horizontal lines in **(C, D)** indicate average bias (continuous line) and 95% confidence levels of agreement (dashed lines).

Data were analyzed using Microsoft Excel 2016 (Microsoft Inc.) and GraphPad Prism 9.3.1 (GraphPad Software). LC-MS absorbance and chromatogram data were exported to Microsoft Excel 2016®. The area under the absorbance peak for the known concentration in the insecticide standard was used to plot the standard curve. The standard curve was then used to determine the insecticide concentration in the unknown samples in g/kg (**Figures 1**, **2A, B**).

Linear regressions depicting the 95% confidence interval bands of the best-fit curve were used to analyze the relationship between LC-MS and XRF data. Pearson’s correlation was used to estimate the degree of correlation between LC-MS and XRF measurements (**Figures 2A, B**). This was appropriate as, even though the data exhibited non-normal distributions, they were continuous, paired, and exhibited similar variances. Bland–Altman methods were used to assess the agreement between individual insecticide measurements from LC-MS and XRF (**Figures 2C, D**).

**Figure 3:**
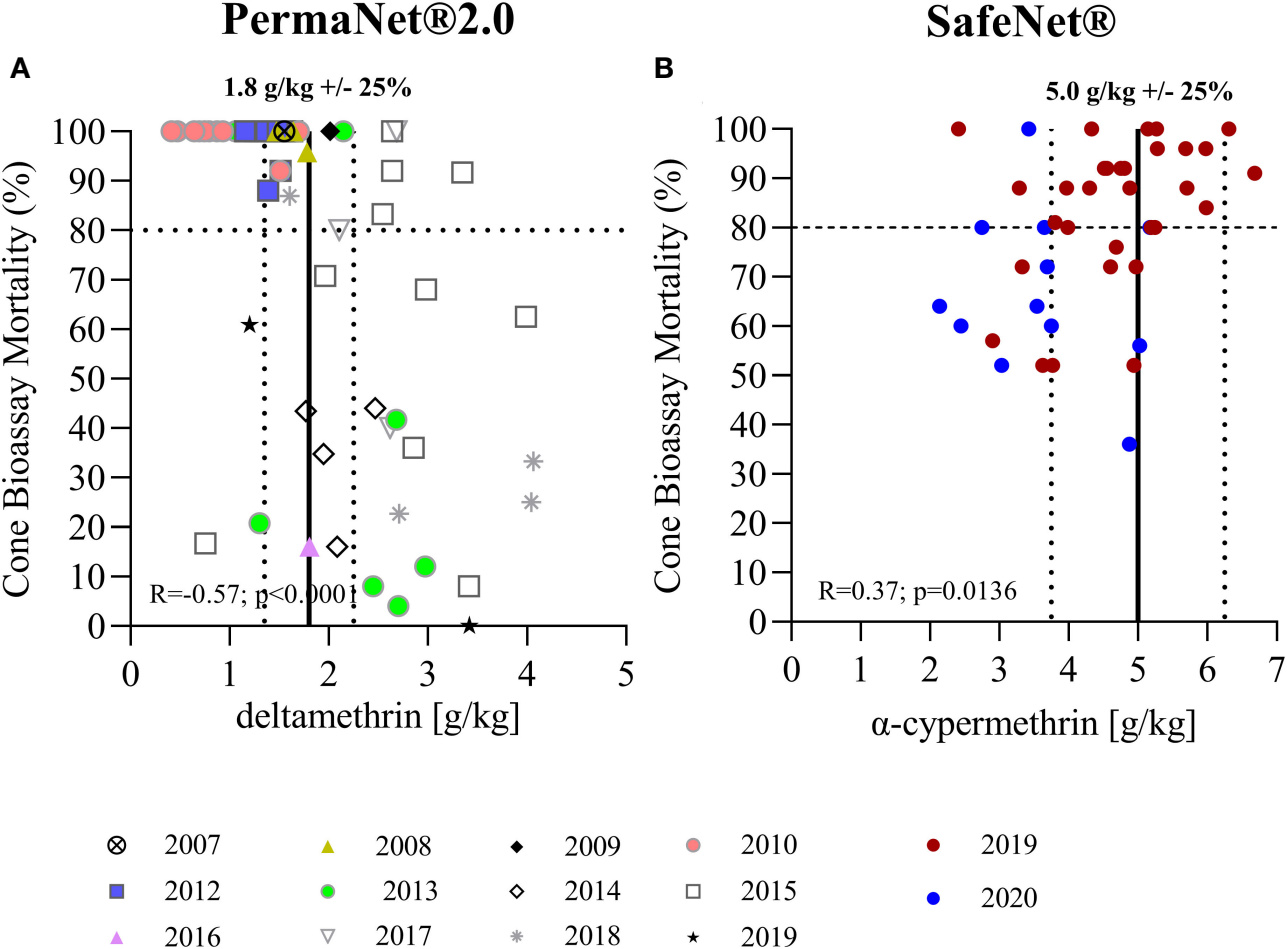
Correlation of total insecticide content measured by LC-MS and bioefficacy. **(A)** Shows the correlation of total AI content as measured by LC-MS with mortality rates observed in standard cone bioassays for PermaNet® 2.0. **(B)** Shows data for SafeNet®. Vertical lines indicate the target AI concentrations as per product label (average ± 25%). The horizontal line indicates the 80% threshold mortality for standard WHO cone bioassays.

The main outcome variable of WHO cone bioassays was 24-h mosquito mortality. Test results were adjusted using “Abbott’s formula” when negative control 24-h mortality was > 0% and ≤ 10%. Linear regression and Pearson’s correlation were used to assess the correlation between bioefficacy and insecticide concentration (**Figure 3**).

Due to the lack of non-Gaussian distribution of the data, non-parametric tests were used to compare insecticide content and bioefficacy between older and newer nets (**Figures 2**, **3**).

## Results

### Liquid chromatography mass spectrometry protocol validation experiments

Solvent volume and extraction time did not systematically affect the experimental outcomes. For all nets used in these validation experiments, the measured deltamethrin concentrations were in the expected range and were between 1.7 g/kg and 1.9 g/kg (**Figures 1A, B**). In all further analyses, 1 mL of methanol was used, and the extraction time was fixed to 10 min. In addition, repeated extractions indicated that the efficiency of the first extraction was > 95% for alpha-cypermethrin (**Figure 1C**). The second extraction resulted in no measurable AI peak using the LC-MS method. These measurements were taken only for alpha-cypermethrin.

### Correlation of insecticide content measurements between XRF and LC-MS methods

XRF and LC-MS insecticide quantification results were compared for both PermaNet® 2.0 and SafeNet® LLINs (**Figures 2A, B**). XRF and LC-MS data were significantly correlated for both products (PermaNet® 2.0: R = 0.78, *p* < 0.0001 and SafeNet®: R = 0.47, *p* = 0.0012, respectively); however, the coefficient of determination was much higher for PermaNet® 2.0 LLINs. Batches of products clustered significantly, indicating differences between specific product batches. In particular, for PermaNet® 2.0 LLINs, this may be due to the wide period of manufacture, dating back to 2007. Older PermaNet® 2.0 nets generally contained fewer AIs (*p* < 0.0001), indicating that AIs may have been gradually lost over time. Analysis of the storage conditions of the ITNs are urgently needed, as they might provide insight into factors that contribute to the loss of AI over time.

The Bland–Altman analysis (presented in **Figures 2C, D**) indicated good agreement between XRF and LC-MS quantification for both products, with no systematic bias and a very close average agreement of the two methods (< 0.05 g/kg). However, the 95% confidence levels of agreement between methods were substantial (± 0.8 g/kg for PermaNet 2.0® LLINs and ± 2.1 g/kg for SafeNet® LLINs) indicating large variation in the agreement for individual samples and limiting the ability of XRF to predict insecticide concentration accurately for individual samples.

### Correlation of insecticide content with bioefficacy

The correlation between the results of standardized WHO cone bioassays and the chemical analysis are shown in **Figure 3**. Strikingly, bioefficacy was lower for more recent (2013–2019) PermaNet® 2.0 samples with a higher total AI content (R = −0.57, *p* < 0.0001). This was unexpected and clearly illustrates that total AI content is not a robust indicator of bioefficacy. In contrast, a positive correlation was observed between bioefficacy and total AI content in the SafeNet® samples (R = 0.37, *p* = 0.0136). This is normally expected; however, not all nets exhibited > 80% mortality, as indicated in the respective WHOPES report underlying prequalification of SafeNet® (WHO). The 2019-SafeNet® had a lower AI content (Mann–Whitney *U* test; *p* = 0.0076) and performed better (Mann–Whitney *U* test, *p* = 0.0041) than 2020-SafeNet®. This difference could be due to the longer period of storage of the 2019 samples or due to differences in the production batches. Given that all nets were stored in air-conditioned storerooms between sampling for bioefficacy and chemical analyses, any further decay in AIs during that period is unlikely. Further studies are required to understand this difference. The comparison of XRF spectroscopy measurements with mosquito mortality data from cone bioassays can be found in **Supplementary Figure S2**.

## Discussion

The present study was aimed at estimating the accuracy of XRF, a field-deployable tool, to quantify not only deltamethrin but also alpha-cypermethrin in LLIN samples. For this, LLIN samples were obtained from consignments of LLINs for mass distribution in PNG. A robust method to quantify insecticide content may be beneficial for researchers and programs, e.g., for use in LLIN durability studies or for in-country quality assurance spot checks. These results illustrate that XRF and LC-MS data are highly correlated, indicating that XRF could serve this purpose. However, the Bland–Altman analyses showed that the agreement between methods in this study was subject to substantial uncertainty. The major source for this uncertainty was that the samples (while derived from the same LLINs) were not exactly the same, i.e., the XRF measurements were done on a different (adjacent) part of the same net from the LC-MS measurements. It is known that individual LLINs can exhibit substantial spatial variation in insecticide content because of the manufacturing process (Skovmand et al., 2021). Therefore, the observed uncertainty is not overly surprising. Despite this limitation, these results showed that even single measurements on different samples from the same LLIN using the two different methods are, on average, very well correlated. Further studies averaging multiple XRF and LC-MS measurements across single LLINs are needed to assess the accuracy of XRF to predict if a product conforms to label specifications.

The CIPAC methods use HPLC in quantifying the pyrethroids and utilize a variety of extraction conditions depending on the insecticide/fiber combination (Green et al., 2009; Jenkins et al., 2013; Smith et al., 2018). In contrast, the method presented here employs a simple extraction, using methanol only as solvent and no heat to prepare samples for LC-MS. The data presented here provide evidence that the method is robust. Neither solvent volume nor extraction time significantly influenced results, and extraction was shown to be complete (> 95%) after one extraction step. However, while this method is appropriate for LLINs that use AI impregnation technologies where all insecticide is present in a coating at the surface of the material (polyester yarns), including PermaNet® 2.0 LLINs, this method is unlikely to be directly transferable to incorporated nets where most of the insecticide is inside the polymer matrix, such as the polyethylene LLINs. Methanol causes the chemical isomerization of deltamethrin (Perschke and Hussain, 1992). Therefore, this study protocol (limited by the availability of equipment) did not allow for an analysis of the deltamethrin isomers present in the samples. Further studies are required to confirm the isomer ratio of deltamethrin in PermaNet® 2.0 LLINs manufactured prior to or in 2012 and after 2012.

The second objective of this study was to correlate bioefficacy results as measured in standardized WHO cone bioassays with total AI content measurements. This was done to explore if total AI content is a robust indicator of bioefficacy in new and unused LLINs. With the most recent (2013–2019) PermaNet® 2.0, the authors observed an inverse correlation between bioefficacy and total AI content, which was unexpected and counterintuitive, indicating that total AI concentration measurements do not always predict bioefficacy well. The reason for the decreased bioefficacy of most of the recent PermaNet® 2.0 LLINs is likely to be related to a change in the coating formulation that caused restricted bioavailability on the net surface, leading to reduced mosquito mortality, as observed in WHO cone bioassays (Bubun et al., 2022).

## Conclusions

This study showed that XRF is a promising field-deployable tool not only for deltamethrin but also for alpha-cypermethrin. Further studies should investigate XRF utility for measuring other insecticides used in the public health spaces (e.g., for indoor residual spraying). This study provided evidence that total AI concentration is not a robust indicator of bioefficacy in new and unused LLIN products, and that bioefficacy tests should be included in predelivery inspections. For PermaNet® 2.0 LLINs, the nets with a high AI content performed poorly in standardized cone bioassays.

## Data availability statement

The original contributions presented in the study are included in the article/**Supplementary Material**. Further inquiries can be directed to the corresponding author.

## Author contributions

MK: Formal Analysis, Investigation, Methodology, Software, Writing – original draft, Writing – review & editing. NB: Investigation, Methodology, Writing – review & editing. DW: Investigation, Methodology, Writing – review & editing. EA: Investigation, Methodology, Writing – review & editing. LT: Writing – review & editing. PJ: Writing – review & editing. ND: Writing – review & editing. ML: Writing – review & editing. TF: Writing – review & editing. SK: Conceptualization, Formal Analysis, Funding acquisition, Investigation, Methodology, Resources, Software, Supervision, Writing – original draft, Writing – review & editing.

## Supplementary material

Supplementary Figure 1 XRF measurement of the insecticide treated nets. A 25 cm × 25 cm net sample was placed on the XRF analyzer, and the shielded chamber was placed onto the net before the measurement was obtained.

Supplementary Figure 2 Correlation of total insecticide content measured by XRF analyzer and bioefficacy. Panel A shows the correlation of total AI content as measured by XRF with mortality rates observed in standard cone bioassays for PermaNet^®^ 2.0 LLINs. Panel B shows data for SafeNet^®^ LLINs.

Supplementary Table 1 Details of PermaNet^®^ 2.0 and SafeNet^®^ LLINs, and LC-MS, XRF, and cone bioassay data.
