# Supplementary figures and images for: Analysis of insecticides in long-lasting insecticidal nets using X-ray fluorescence spectroscopy and correlation with bioefficacy
Source: Front Parasitol. 2023 Oct 11;2:1258429. doi: 10.3389/fpara.2023.1258429 (PMC11732067; doi:10.3389/fpara.2023.1258429)

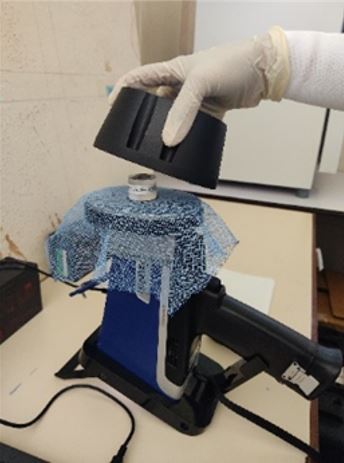

Supplement: Supplementary Figure 1 — XRF measurement of the insecticide treated nets. A 25 cm × 25 cm net sample was placed on the XRF analyzer, and the shielded chamber was placed onto the net before the measurement was obtained. [file Image_1.jpeg]

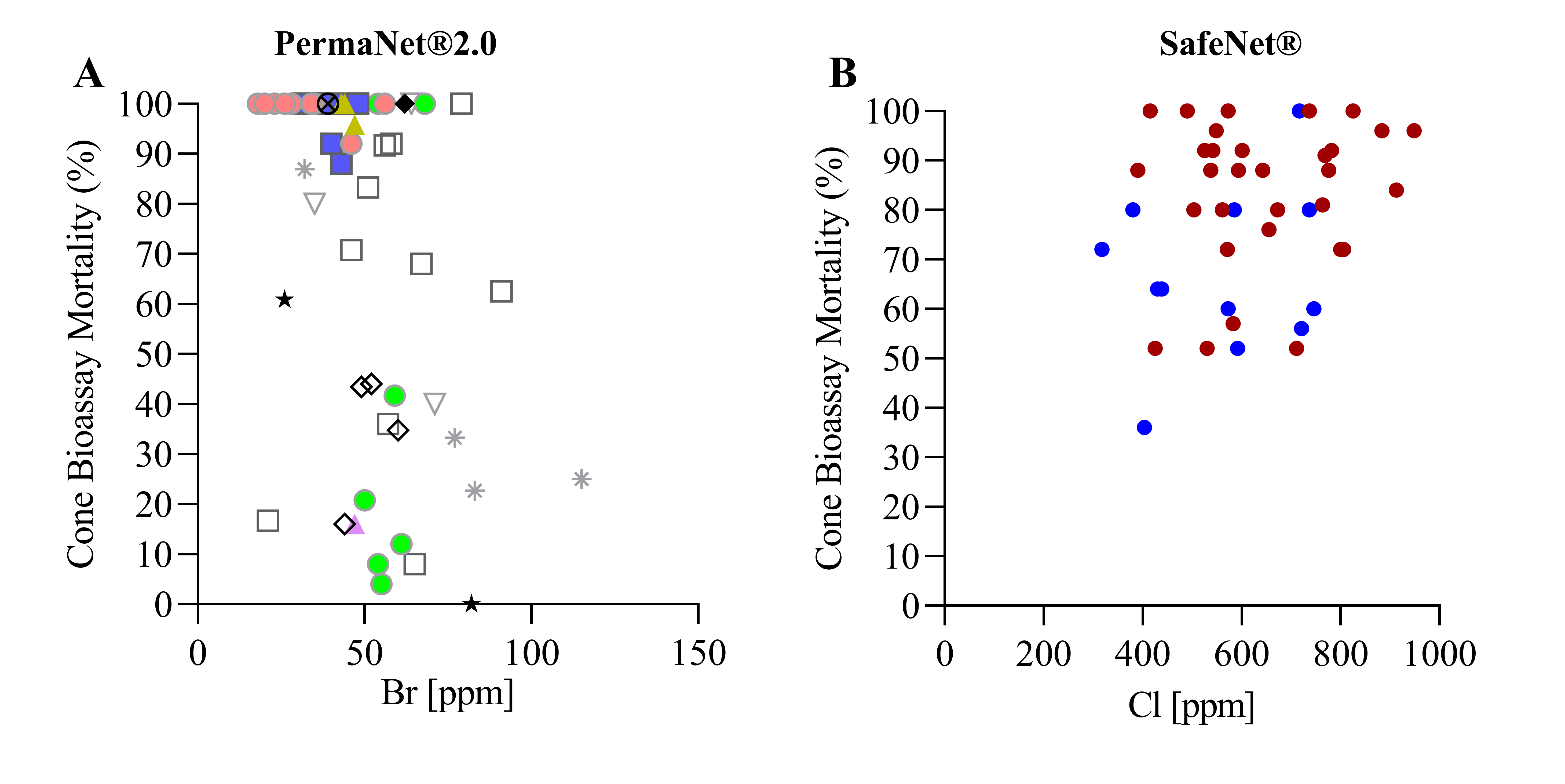

Supplement: Supplementary Figure 2 — Correlation of total insecticide content measured by XRF analyzer and bioefficacy. Panel A shows the correlation of total AI content as measured by XRF with mortality rates observed in standard cone bioassays for PermaNet® 2.0 LLINs. Panel B shows data for SafeNet® LLINs. [file Image_2.tif]
